# Cooperative Sorption on Porous Materials

**DOI:** 10.1021/acs.langmuir.1c01236

**Published:** 2021-08-19

**Authors:** Seishi Shimizu, Nobuyuki Matubayasi

**Affiliations:** † York Structural Biology Laboratory, Department of Chemistry, University of York, Heslington, York YO10 5DD, United Kingdom; ‡ Division of Chemical Engineering, Graduate School of Engineering Science, Osaka University, Toyonaka, Osaka 560-8531, Japan

## Abstract

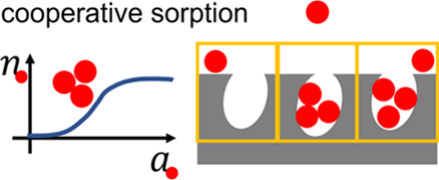

The functional shape of a sorption isotherm is determined by underlying molecular interactions. However, doubts have been raised on whether the sorption mechanism can be understood in principle from analyzing sorption curves via a range of competing models. We have shown recently that it is possible to translate a sorption isotherm to the underlying molecular interactions via rigorous statistical thermodynamics. The aim of this paper is to fill the gap between the statistical thermodynamic theory and analyzing experimental sorption isotherms, especially of microporous and mesoporous materials. Based on a statistical thermodynamic approach to interfaces, we have derived a cooperative isotherm, as a generalization of the Hill isotherm and our cooperative solubilization model, without the need for assumptions on adsorption sites, layers, and pore geometry. Instead, the statistical characterization of sorbates, such as the sorbate-interface distribution function and the sorbate number distribution, as well as the existence of statistically independent units of the interface, underlies the cooperative sorption isotherm. Our isotherm can be applied directly to literature data to reveal a few key system attributes that control the isotherm: the cooperative number of sorbates and the free energy of transferring sorbates from the saturated vapor to the interface. The sorbate–sorbate interaction is quantified also via the Kirkwood–Buff integral and the excess numbers.

## Introduction

Microporous and mesoporous materials,^1,2^ such as activated carbons,^3−5^ porous silica,^6^ and metal–organic frameworks,^7−9^ are powerful adsorbents. They are important not only because of their many industrial applications but also because of the challenges they pose to understanding their sorption capacities from a molecular basis.^1−9^

One approach is to understand the functional shape of an isotherm based on the underlying molecular interactions influenced by interfacial geometry and pore sizes.^1−9^ Here, we first clarify why understanding cooperative sorption isotherms has been particularly challenging despite, or because of, the many isotherm models that have been proposed.^4,10,11^ Thereafter, we will show how these difficulties can be overcome via statistical thermodynamics.

### Sorption Isotherm Models

There are, broadly speaking, three classes of isotherm models: empirical, semi-empirical, and physical.^12,13^ The empirical models can fit experimental data; yet, since they are not based on a physical basis, they cannot be used to understand sorption mechanisms. Examples include the Sips model^14^ used for gate-opening adsorption.^7−9^ The semi-empirical models start from some plausible physical principles, such as those based on Polanyi’s adsorption potential,^15−17^ leading to the Frenkel–Halsey–Hill (FHH)^18−21^ as well as the Dubinin–Astakhov^22^ and Dubinin–Radushkevich^22−26^ models used for microporous carbons. However, as admitted by Dubinin, ″it became more and more obvious that the initial principles of the potential theory have no physical meaning for adsorption in micropores″.^25^ Since our goal is to gain insights into molecular mechanism through the analysis of sorption isotherms, we shall focus on the physical isotherm models. Historically, understanding sorption based on physical models fitted to experimental data has been limited by the fact that multiple isotherm models, with different assumptions on sorption mechanisms,^27−32^ can fit an experimental isotherm equally well.^33^ The goodness of the fit alone cannot be used to conclude the superior realism of one model over the rest.^33^

### Multilayer Adsorption Models and Their Limitations

Many of the approaches to adsorption based on a physical model have their roots in the monolayer adsorption model by Langmuir,^34^ which was generalized later to multilayer adsorption as the Brunauer–Emmett–Teller (BET) model^1,35^ and extended further into the Guggenheim–Anderson–de Boer (GAB) model,^36−38^ to incorporate the difference between the second layer and outer layers. These models presuppose adsorption on planar surfaces or at least one single binding constant for each different class of layers (e.g., first, second, and outer layers). BET and GAB models assumed an infinite number of layers.^12,13^ Assuming a finite number of layers instead of infinity has led to the recent ζ-isotherm,^39^ which captures the cooperative sorption behavior,^10,39^ based on a generalization of (T.L.) Hill’s re-derivation of BET and GAB isotherm from a grand canonical ensemble.^40^ The basic assumption was the binding of clusters on multiple, independent adsorption sites on a surface.^40^ However, whether there are well-defined and independent binding sites on a surface is still an assumption, just like the number of layers assumed in the original derivation.

### Binding Models and Their Limitations

Cooperativity in ligand binding to proteins was modeled by (A.V.) Hill as early as 1910.^41^ Its generalization by Adair,^42^ Klotz,^43,44^ Koshland, and Wyman^45−47^ led to the concept of cooperative binding, expressed via the binding polynomial.^40,44,47−50^ The binding polynomial is founded on the grand canonical partition function. Yet, in practice, it was interpreted as the successive and stepwise binding of multiple ligands on the binding sites.^40,44,47−50^ Such an assumption reflects the reality for protein–ligand binding with well-defined binding sites. However, the application of the binding polynomial to protein denaturants and stabilizers (that work through competitive or ″preferential″ solvation of water and denaturant or water and stabilizer)^51−53^ caused difficulties, confusions, and controversies^54−56^ because the ″binding sites″ and ″binding constants″ for denaturants and stabilizers on proteins cannot be defined with clarity.^51−53^ The resolution came by abandoning the binding-based view of solvation altogether, replacing it with the fluctuation theory, to capture the weak, nonspecific, and nonstoichiometric interaction between cosolvents and proteins.^57−60^ In addition, borrowing the concepts from adsorption to apply directly to solvation (and vice versa) while neglecting the difference in the thermodynamic degrees of freedom caused further confusion.^59,61,62^

### Sorption on Nonplanar and Porous Surfaces

Two major difficulties face the approaches based on simple physical models that assume binding/adsorption sites and layers. Even in the simpler sorbents that obey the BET model, the ″BET surface area″^1,12,13^ is dependent on the sorbate gases.^63^ Moreover, capillary condensation has been considered to be the driving force of the large gradient of isotherms for porous systems.^13^ The key, according to the Kelvin equation, is the condensation of sorbate vapor in the pore at a lower critical pressure than in the bulk.^13^ This explanation was refined using the thermodynamic stability theory for nano- and mesoscale systems,^64^ according to which the sharp change of sorption isotherms has been attributed to the sorbate number fluctuation reaching the size scale of the pore.^65^ Such a cooperative phenomenon may not be captured sufficiently by binding sites and layers even under the cooperative binding, especially in the systems that contain a very small amount of surface functional groups.^66^ Such considerations necessitate an approach to sorption that does not depend on model assumptions. The most general foundation for adsorption is the Gibbs isotherm,^67−69^ which was derived from a triad of the Gibbs–Duhem equations. However, the application of the Gibbs isotherm was limited by the implementation of the dividing surface that employs the concentration profile.^65,70^ To define the concentration profile, an axis representing a distance from the interface is necessary.^65,70^ However, such a coordinate is hard to implement for porous and inhomogeneous surfaces.

### The Generalized Gibbs Isotherm

The difficulties of the multilayer and binding models, as summarized above, can be overcome by the following strategies: (i) generalizing the Gibbs adsorption isotherm based only on the basic principles of statistical thermodynamics;^65^ (ii) generalizing the Gibbs dividing surface condition to be free of concentration profiles, making it applicable to any surface geometry even in the presence of cavities and crevices;^65^ and (iii) introducing the interfacial local subsystem based on the finite-ranged nature of the interfacial effect, thereby enabling to approach sorption from the statistical thermodynamically defined interactions, such as the distribution of sorbates and the sorbate–sorbent and sorbate–sorbate correlations, instead of assuming binding constants and sites.^65^

### Cooperativity from the Fluctuation Theory

Based on the generalized Gibbs isotherm, a theory of sorption was formulated in a manner analogous to the fluctuation theory for solvation. We have shown that sorbate–sorbate interaction, which has been considered to play an important role in the functional shape of an isotherm,^4,5,71−73^ can be quantified directly from an isotherm’s derivative.^65^ (We emphasize that the sorbate–sorbate interaction, which takes place at the interface, is mediated by the interface.) From the experimental data on water vapor adsorption on microporous and mesoporous carbons,^3,4,66,74^ the underlying sorbate–sorbate interaction has been quantified. (This is analogous to the cosolvent–cosolvent interaction, when enhanced by the solute, which leads to the cooperative onset of solubilization.^61,75−77^)

### Need for a Cooperative Isotherm Equation from the Fluctuation Theory

In contrast to some previous physical isotherm models based on different assumptions,^10,78−80^ our statistical thermodynamic theory, despite its rigorous nature, was unable to reproduce the isotherm curve for cooperative sorption. Its success was limited to analyzing the gradient of an isotherm only at its cooperative onset.^65^ However, our recent progress provides the tools for constructing a cooperative isotherm curve based on statistical thermodynamics. First, the general isotherm, encompassing the classical models such as Langmuir,^34^ BET,^1,35^ and GAB,^36−38^ can be constructed statistical thermodynamically based on multiple body correlations between sorbates at the interface.^70^ Second, the sigmoidal increase of solubilization in the presence of hydrotropes can be modeled statistical thermodynamically,^81^ which can describe the sigmoidal shape of solubilization curves.^82,83^ In this paper, these two theories will be extended further for a cooperative sorption theory.

Thus, the goal of this paper is to construct a function for cooperative sorption isotherm. Instead of the binding polynomials, our theoretical foundation is the statistical thermodynamic generalization of the Gibbs isotherm. The resultant cooperative isotherm is mathematically analogous to the (A.V.) Hill model of cooperative binding. However, the underlying molecular mechanisms are different. While the cooperative binding model assumes the binding of sorbates on the well-defined binding sites modeled by the binding constants,^40,44,47−50^ our cooperative sorption theory is founded on a statistical nature of interactions: the local–bulk division of the interface from the sorbate–interface distribution function (″Theory″ section), implementation of statistically independent units of the interface, and the sorbate number distribution in those interfacial units as the basis of cooperativity (″Results and Discussion″). Unlike the binding model, our theory is applicable to both specific and nonspecific interactions between sorbate and interface that have been incorporated in a fully statistical manner. The cooperative isotherm will be applied to fit the water sorption isotherms on porous carbons.

## Theory

### Statistical Thermodynamics of Sorption

#### The Generalized Gibbs Isotherm

Our goal is to derive a sorption isotherm that can fit the experimental isotherm of porous surfaces directly from the principles of statistical thermodynamics. Let us consider the interface between the phases *I* and *II*. Phase *I* is composed of the sorbent (molecular species 1), and phase *II* is composed of the sorbate (molecular species 2). The interface does not need to be planar, which is the advantage of our generalized statistical thermodynamic approach.^65^ The entire system, denoted by *, contains *I* and *II*, as well as the interface between them.^12,67,69^ The thermodynamic effect of the interface is the difference between the entire system (*, with the interface) and the reference systems (*I* + *II*, without the interface).^12,65,67,69^

The conventional derivation of the Gibbs isotherm via the Gibbs–Duhem equations employs the concentration profile.^12,13,67,69^ Because of the need for a (clearly defined) coordinate for the concentration profile to introduce the Gibbs dividing surface, this approach introduces an unnecessary restriction to planar interfaces.^65^ Instead, we start from the following general thermodynamic relationship without any assumptions, applicable to any surface geometry and porosity:^62,65^
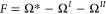
1in terms of the difference in the thermodynamic function (Ω = –*PV*) between the entire system (*) and the two reference systems (*I* + *II*) under the conservation of volume.^62,65^ Here, instead of the product of γ (surface free energy) and *A* (surface area), we use the total interfacial free energy, *F*, because of the difficulty in defining the surface area with accuracy when the interface is not planar, e.g., for microporous and mesoporous systems. The three systems are open to both species.

Now, we incorporate the Gibbs dividing surface without the restriction of concentration profiles. This can be achieved via the Legendre transformation, converting the thermodynamic function Ω (open to species 1 and 2) to *Y* = Ω + μ_1_*N*_1_ (open to species 2 but closed to 1), as
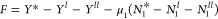
2where μ_1_ is the chemical potential of species 1.^65^ Imposing the condition *N*_1_^*^ – *N*_1_^*I*^ – *N*_1_^*II*^ = 0 on eq 2 is equivalent to introducing the Gibbs dividing surface but without the concentration profile. Equation 2 now becomes
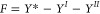
3aEquation 3a applies to any surface geometry and porosity. Using the corresponding partition functions for the semi-open systems, Γ*, Γ^I^, and Γ^II^, eq 3a can be rewritten as
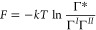
3bEquation 3b is our fundamental relationship. Differentiating eq 3b with respect to ln*a*_2_ (*a*_2_ is the activity of sorbate), through elementary statistical thermodynamic calculus, yields the generalized Gibbs adsorption isotherm^65^
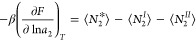
4where ⟨ ⟩ denotes ensemble average. Equation 4 is applicable to any surface geometry even in the presence of cavities and crevices.^65^

#### The Interfacial Subsystem

The effect of an interface is confined within a finite distance from the surface, which is our basic postulate.^65^ Let v* be the volume contained within this finite distance, which we call the interfacial local subsystem. This distinction has a statistical thermodynamic basis. Consider the interface–sorbate distribution function.^84^ The local subsystem contains the maxima and minima of the distribution function, whereas the bulk region is characterized by its convergence. Under this postulate, we have shown previously that the Gibbs adsorption isotherm can be expressed as^65,81^
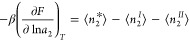
5in terms of the difference in number between the interfacial subsystem, ⟨*n*_2_^*^⟩, and the reference subsystems *I* and *II*, ⟨*n*_2_^*I*^⟩ and ⟨*n*_2_^*II*^⟩, that have the volumes *v^I^* and *v^II^*.^65^ For each system (*, *I*, and *II*), the partition function of the semi-open system, Γ(*T*, *V*, *N*_1_, μ_2_), can be expressed relative to the partition function of pure species 1, Γ(*T*, *V*, *N*_1_, ∞ ), as
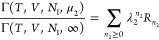
6where λ_2_ = *e*^βμ_2_^ and *R*_*n*_2__ is the partition function with *n*_2_ sorbates in the subsystem of volume *v*. Equation 6 was derived by distributing the molecules in the system between the local subsystem and the bulk.^65,81^ What is important in eq 6 is that *R*_*n*_2__ is a quantity pertaining to the interface, which will serve as the basis for our sorption isotherm in ″Results and Discussion″.

### A Statistical Thermodynamic Foundation for Sorption Isotherms

#### Vapor Sorption Isotherms

We have derived the generalized Gibbs adsorption isotherm, expressed in terms of the local subsystems (eq 5). Here, we derive a sorption isotherm for microporous and mesoporous materials directly from statistical thermodynamics. Our starting point is the statistical thermodynamic relationship for the mean number of sorbate molecules in the local subsystems, ⟨*n*_2_^*^⟩, ⟨*n*_2_^*I*^⟩, and ⟨*n*_2_^*II*^⟩, expressed in terms of the respective partition functions,
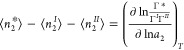
7So far, our discussion has been general, applicable to any interfaces.

In the present work, we focus on gaseous adsorbates, for which there is a wealth of experimental data on the sorption on microporous and mesoporous materials. The left-hand side of eq 7 is determined by excess densities of species 2 in the entire system * from those in the reference systems *I* and *II*. In our treatment, the excess is assumed to be localized within a finite distance from the interface, and in this assumption, ⟨*n*_2_^*^⟩ – ⟨*n*_2_^*I*^⟩ – ⟨*n*_2_^*II*^⟩ reflects only the contributions from the interfacial region. We further suppose that ⟨*n*_2_^*^⟩ ≫ ⟨*n*_2_^*I*^⟩ and ⟨*n*_2_^*^⟩ ≫ ⟨*n*_2_^*II*^⟩ hold when the system is set to the interfacial region. The former corresponds to the absence of adsorption into phase *I*, and the latter means that the density in the vapor is negligible compared to that in the interfacial region. In the following, we restrict our attention to the interfacial region, and with the above two suppositions, eq 7 is simplified as
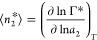
8Equation 8 is our basic relationship, from which sorption isotherms can be derived. From now onward, we will drop * for simplicity unless otherwise noted.

#### The Sorption Polynomial

Here, we introduce the ″sorption polynomial″ as a generalization of the binding polynomials. The sorption polynomial is the series expansion of Γ* in eq 8. To carry this out, we will generalize the elegant approach proposed by McMillan and Mayer^85^ as has been done before in our cooperative solubilization theory.^81^ Here we consider *N*_2_^b^ = *N*_2_ – *n*_2_, the number of adsorbates outside the local subsystem, for both the entire system (*) and the reference system (II). Following McMillan and Mayer,^85^ we consider the *N*_2_^b^ → 0 limit of eq 6. (In our definition of the local subsystem, *v* represents the ranges of correlations among the interface and sorbate molecules. Hence, putting *N*_2_^b^ → 0 is equivalent to ignoring the contribution from the sorbate molecules outside of the correlation range.)

At this limit, all the terms of *R*_*n*_2__ become a constant, and eq 6 can be considered as the polynomial expansion of  around *a*_2_ = 0. This is true for both  and . In the latter, especially, neglecting ⟨*n*_2_^*II*^⟩ in eq 8 is equivalent to ignoring all the terms except for *n*_2_ = 0, leading to . Since Γ*(*T*, *V*, *N*_1_, ∞ ) and Γ^II^(*T*, *V*, *N*_1_, ∞ ) are both independent of *a*_2_ and *R*_*n*_2__ is independent of *a*_2_, eq 6 yields
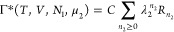
9awhere *C* is a constant, which will vanish when differentiated with respect to ln*a*_2_. Since *C* does not affect the sorption isotherm, we introduce the following ″sorption polynomial″:
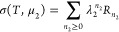
9bFrom the sorption polynomial, the isotherm can be derived using the following equation, which is a modification of eq 8:
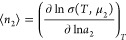
9c

#### Extensive Nature of Sorption Isotherms and Polynomials

Here we present a detailed statistical thermodynamic formalism for the sorption polynomials based on statistically independent interfacial units. Such a task is more challenging than dividing the total partition function of an ideal gas into statistically independent units, i.e., molecular partition functions.^86^ The additional difficulty for cooperative sorption comes from the need to consider multiple sorbate molecules and their statistical distribution that interact with a porous sorbent. As the first step, we consider the extensive nature of the sorption isotherms and sorption polynomials. Distinguishing extensive and intensive thermodynamic quantities provides valuable insights into solvation and adsorption.^62,87^ The amount of adsorbate is an extensive thermodynamic quantity that scales with the amount of adsorbent. Such an extensive nature is employed in the experimental measurements of sorption isotherms that are usually reported per unit quantity of sorbent. Suppose that we scale the interface by λ times while keeping the thickness of the interfacial subsystem constant. This leads to a λ-fold increase of sorption, and ⟨*n*_2_⟩ scales to *λ*⟨*n*_2_⟩. Here, λ can either be greater than 1 or smaller than 1. If λ < 1, the local interfacial subsystem, introduced in the ″Theory″ section, is subdivided into *N* = 1/λ subsubsystems.

#### Implementation of the Intensive ″Unit Interface″

The extensive nature of ⟨*n*_2_⟩, via eq 9c, is equivalent to the extensive nature of lnσ(*T*, μ_2_). This means that the sorption polynomial σ(*T*, μ_2_) can be expressed as
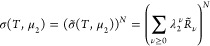
10in terms of σ̃(*T*, μ_2_), which will be referred to as the sorption polynomial of the interfacial unit, and *R̃*_ν_ is the corresponding coefficient. A general sorption isotherm can be obtained by combining eqs 10 and 9c as
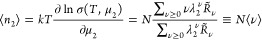
11Here we introduce
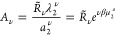
12with μ_2_^*o*^ as the standard chemical potential of the sorbate and rewrite eq 11 explicitly in terms of the sorbate activity, *a*_2_. We obtain
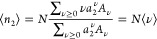
13The physical meaning of eqs 10–13 can be understood from the general relationship between ⟨*n*_2_⟩ and its fluctuation, ⟨*n*_2_^2^⟩ – ⟨*n*_2_⟩^2^,
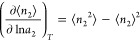
14aSubstituting eq 13 into eq 14a, we obtain
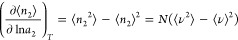
14bshowing that the interfacial subsystem is composed of *N* statistically independent unit interfaces. Note that ⟨*n*_2_^2^⟩ – ⟨*n*_2_⟩^2^ is *O*(*n*_2_) (*O* is Landau’s symbol, signifying ″in the same order of″). Now we postulate that the unit interface does not scale with system size. Under this condition, *N* is *O*(*n*_2_), whereas ⟨*ν*^2^⟩ – ⟨*ν*⟩^2^ is *O*(1). This means that the isotherm, expressed in terms of the unit interface via eq 13, refers to the scaling of the following form:
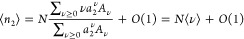
15

Now we show that the functional form of ln⟨ν⟩ can be determined uniquely from the excess number of sorbates around a probe sorbate molecule, *N*_22_, defined as^65^
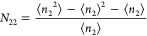
16aNote that *N*_22_ is an intensive quantity, reflecting sorbate–sorbate interaction, which does not scale with system size. *N*_22_ is linked to the sorption isotherm, i.e., ⟨*n*_2_⟩ as a function of *a*_2_, via the following general statistical thermodynamic relationship:
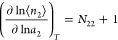
16bSince *N*_22_ refers to sorbate–sorbate interaction, it is an intensive quantity. Substituting eq 13 into eq 16b yields
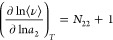
16cThe functional form of ln⟨ν⟩ can be determined uniquely by integrating eq 16c using the *a*_2_ dependence of *N*_22_ that can be calculated from the experimental isotherm. This means that the functional form of ⟨ν⟩ can be determined uniquely from an experimental isotherm except for a multiplicative constant that does not affect the reality of the structure of sorbates expressed via eq 16b.

Equation 13 is our general isotherm. The only postulates introduced in deriving eq 13 are (i) that the effect of an interface is confined within a finite distance and (ii) that the interface can be expressed in terms of *N* statistically independent unit interfaces. All the parameters in eq 13 have a clear physical meaning: ν is the number of sorbates in a unit interface. *A*_ν_ is related to the free energy of transferring ν sorbate molecules from the standard state (saturated vapor) to the unit interface via
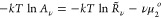
17This means that  is the work required to transfer a sorbate (that behaves within ν sorbates that sorb together) from the saturated vapor to the interface. This interpretation is reminiscent of Polanyi’s ″adsorption potential″ whose physical meaning has been questioned.^17,25^ The difference is that this work, , is dependent on ν and, unlike the adsorption potential, does not refer to any single sorbate molecule found at the interface. Most importantly, contrary to the successive binding model, the free energy change, which accompanies the transfer of ν sorbates (eq 17), is a statistical thermodynamic concept, applicable regardless of the specificity and strength of sorbate–sorbent and sorbate–sorbate interactions.

Thus, we have established a statistical thermodynamic isotherm. Unlike the successive binding or multiplayer adsorption models, eq 13 was derived directly from the principles of statistical thermodynamics via the locality of the interfacial effect and the extensive nature of sorption isotherms.

## Results and Discussion

### Cooperative Adsorption Theory

Here we apply our general isotherm (eq 13) to moisture adsorption on porous surfaces. Instead of introducing pores explicitly into the model, we introduce several postulates to capture sorption cooperativity.

Experimental isotherms exhibit a sigmoidal functional shape, which shows there is cooperativity at work.^3,4,66,74^ Such sigmoidal isotherms can be captured by assuming that the dominant contribution to the sorption polynomial comes from the *m*th order (*a*_2_^*m*^) term. This means that *m* sorbates sorb cooperatively onto the unit interface. Appendix A presents a detailed justification, based on sorbate number distribution within a unit interface, to neglect all the other terms except for the *m*th order term. The zeroth- and first-order terms must also be kept such that the *a*_2_ → 0 limit satisfies Henry’s law.^12,13,17,88^ Therefore, eq 13 becomes
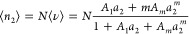
18

In the ″Theory″ section, we have shown that *m* is determined uniquely from the excess sorbate number and that ln⟨ν⟩ can be determined uniquely from an isotherm. Here, fitting eq 18 to an experimental isotherm enables a unique determination not only of *m* but also of *A*_1_ and *A_m_* due to the presence of 1 in the denominator. We further postulate the existence of a statistically independent unit interface that is microscopic in size. Consequently, the number of adsorbate molecules involved in the series of eq 13 is microscopic, and *m*, *A*_1_, and *A_m_* in eq 18 are at molecular scales. Their values reflect the nature of the independent unit interface and its interactions with adsorbates. However, whether this unit interface corresponds to a pore (if the individual pores are statistically independent) or multiple pores (if they behave as the statistically independent minimum unit) cannot be determined from this postulate alone. Note that if a nonlinear increase of isotherm at low *a*_2_ cannot be captured by eq 18, more terms may be incorporated. Furthermore, *m* values determined from fitting eq 18 to experimental data may not be integers because the adsorbate number distribution has been simplified (Appendix A).

Here, the interpretation of −*kT* ln *A_m_*, as the work of transferring *m* sorbates from saturated vapor to the microscopic unit interface (see ″Theory″), is useful when we apply eq 18 to analyze sorption on porous materials. Even though it is possible to express *A_m_* as the product of successive binding constants (Appendix B), capillary condensation is the driving force for a large gradient of sorption isotherms rather than stoichiometric binding on binding sites. Capillary condensation, according to the thermodynamic stability theory for nano- and mesoscale confinement,^64^ is *N*_22_ + 1 in eqs 16b and 16c reaching the size scale of the pore. This can be captured by the *a*_2_^*m*^ term in eq 18, which allows a nonstoichiometric interpretation of −*kT* ln *A_m_*.

All the parameters in eq 18 can be determined by fitting it to experimental isotherm data. Nonlinear fitting routines are usually capable of carrying out regression. Appendix C presents the mathematical expression for eq 18 when the ″coverage ratio″, θ, is used to report experimental sorption data. Appendix D shows how the parameters in eq 18 can be determined from linearized plots if the input parameters are needed for nonlinear regression.

Now we apply our theory (eq 18) to fit the experimental isotherms of water vapor on phenol-resin (PHE)-based and pitch-based (PIT) hydrophobic activated carbon fibers (ACFs) that have been measured by Nakamura et al.^66^ The pore widths of PHE ACFs are 0.5 and 0.6 nm, respectively, which will be denoted as PHE5 and PHE6; the pore widths of PIT ACFs are 0.6, 1.0, and 1.1 nm, respectively, which will be denoted as PIT6, PIT10, and PIT11.^66^ In these examples, the adsorption and desorption proceed through metastable states with long lifetimes. This is the cause of hysteresis, and the experimental fitting is done for the adsorption data as a common practice. When the physical isotherm models are used, it is (implicitly) assumed that a statistical thermodynamic formulation can be utilized for long-lived metastable states. In our analyses, the statistical thermodynamic theory is employed for both the adsorption and desorption processes. This means, in turn, that the same assumption as the physical models (as discussed above) is adopted in our analysis here.

**Figure 1:**
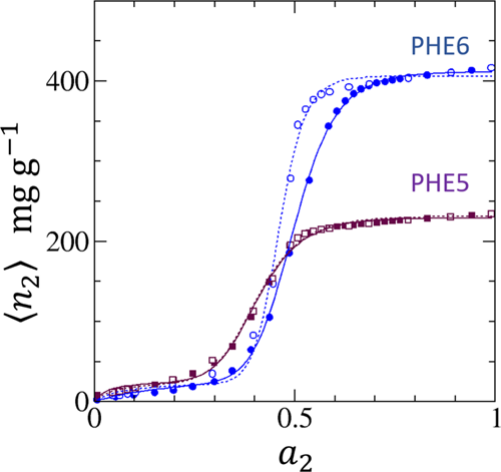
Fitting of experimental adsorption (filled) and desorption (open) isotherms of water vapor on hydrophobic activated carbon fibers using the data by Nakamura et al.^66^ for the phenol-resin (PHE)-based hydrophobic activated carbon fibers, PHE5 (maroon) and PHE6 (blue), with pore widths of 0.5 and 0.6 nm, respectively. The solid lines are the fitting of eq. 18 for the adsorption isotherms and the dotted lines for the desorption isotherms. The fitting parameters are summarized in Table 1.

**Figure 2:**
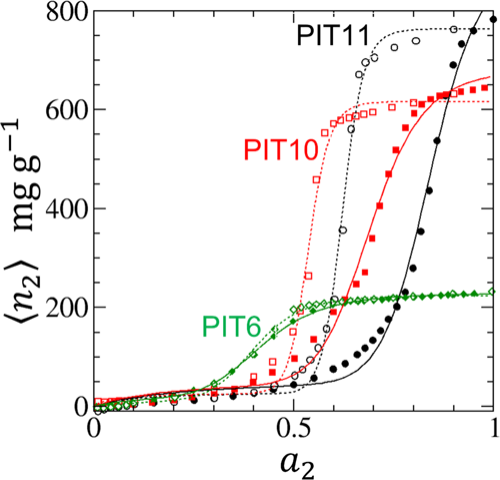
Fitting of experimental adsorption (filled) and desorption (open) isotherms of water vapor on hydrophobic activated carbon fibers using the data by Nakamura et al.^66^ for the pitch-resin (PIT)-based hydrophobic activated carbon fibers, PIT6 (green), PIT10 (red), and PIT11 (black), with pore widths of 0.6, 1.0, and 1.1 nm, respectively. The solid lines are the fitting of eq 18 for the adsorption isotherms and the dotted lines for the desorption isotherms. The fitting parameters are summarized in Table 1.

Figure 1 shows that eq 18 fits the water vapor adsorption and desorption isotherms on PHE5 and PHE6 successfully. The fitting parameters can be found in Table 1. Figure 2 also shows that eq 18 can fit the adsorption and desorption isotherms PIT6, PIT10, and PIT11, with more accuracy for smaller pore widths. The desorption isotherms involve larger *m* than the adsorption counterparts. Table 1 shows that *A*_1_ is small in magnitude compared to *A_m_*, showing that the cooperativity contribution from the *a*_2_^*m*^ term dominates the region of *a*_2_ in which the isotherm exhibit a sigmoidal behavior. Note that the *m* values determined here are not integers. This is not surprising considering that eq 18 is based upon an approximation in Appendix A that simplified the distribution in the adsorbate numbers that sorb cooperatively. Hence, for example, *m* = 8.29 for PHE5 in Table 1 indicates that the cooperative effect is operative around *m* = 8 and 9. The successful fit of the experimental data shows that the onset of cooperative adsorption is captured by eq 18 by the absence of the terms below *a*_2_^*m* – 1^.

**Table 1:** The Fitting Parameters for Eq. 18 for the Experimental Isotherms of Water Vapor on Phenol-Resin (PHE)-Based and Pitch-Based (PIT) Hydrophobic Activated Carbon Fibers (ACFs) Measured by Nakamura et al.^66^^a^

	*N* (mg g^–1^)	*A*_1_	*A_m_*	*m*
PHE5 adsorption	27.7	29.1	2.193 × 10^4^	8.29
PHE5 desorption	28.5	17.8	1.252 × 10^4^	8.13
PHE6 adsorption	40.4	3.94	3.510 × 10^3^	10.2
PHE6 desorption	26.9	13.1	8.395 × 10^5^	15.1
PIT6 adsorption	36.2	4.87	6.760 × 10^2^	6.32
PIT6 desorption	29.9	2.84	1.917 × 10^3^	7.51
PIT10 adsorption	60.8	4.81	2.620 × 10^2^	11.2
PIT10 desorption	28.4	20.2	8.331 × 10^6^	21.7
PIT11 adsorption	62.5	3.83	4.973 × 10^1^	14.5
PIT11 desorption	30.9	9.99	8.790 × 10^5^	24.7

a The pore widths for PHE5 and PHE6 were 0.5 and 0.6 nm, respectively, whereas those for PIT6, PIT10, and PIT11 were 0.6, 1.0, and 1.1 nm, respectively.^66^ The unit for *N* is mg of sorbate per g of sorbent.^66^

#### Excess Sorbate Numbers

**Figure 3:**
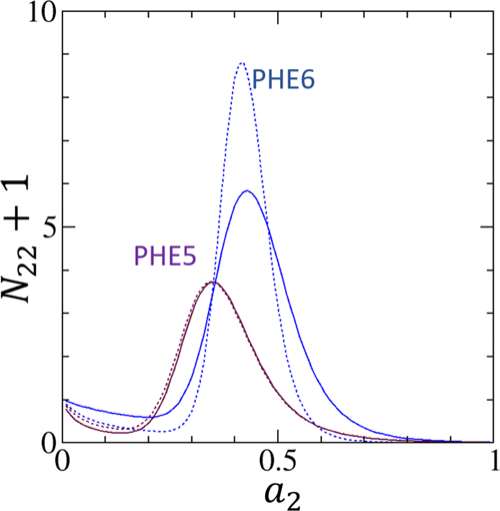
The sorbate cluster number, *N*_22_ + 1, calculated for the adsorption (solid) and desorption (dashed) isotherms of water vapor on PHE5 (maroon) and PHE6 (blue) calculated from eq 19. For the parameters for eq 19, see Figure 1 and Table 1.

**Figure 4:**
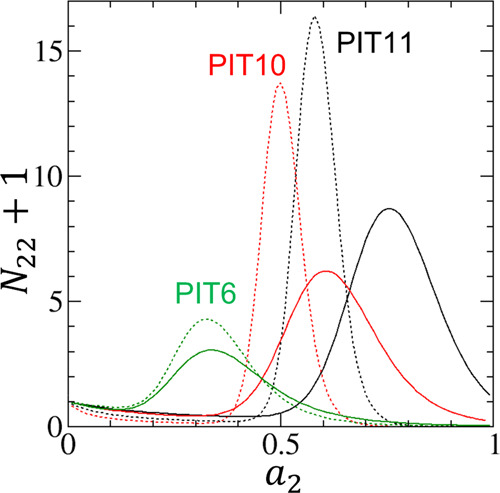
The sorbate cluster number, *N*_22_ + 1, calculated for the adsorption (solid) and desorption (dashed) isotherms of water vapor on PIT6 (green), PIT10 (red), and PIT11 (black) calculated from eq 19. For the parameters for eq 19, see Figure 2 and Table 1.

Based on the statistical thermodynamic cooperative isotherm (eq 18), let us calculate the excess number of sorbates around a probe sorbate molecule. The excess number of sorbates around a probe sorbate molecule, *N*_22_, defined by eq 16a,^65^ can be calculated from the sorption isotherm (eq 18) as
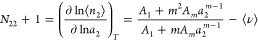
19We have calculated *N*_22_ + 1 both for the PHE and PIT types of porous carbons, shown in Figures 3 and 4, respectively.

Both *m* and *N*_22_ refer to the number of sorbates involved in the sorption process. While *m* is a constant by definition, *N*_22_ is dependent on *a*_2_, exhibiting a peak as seen in Figures 3 and 4 for all the sorbents analyzed, and the peak values of *N*_22_ are generally smaller than *m* (Table 1). *m* refers to the number of sorbates that come into the unit interface. Upon sorption, sorbates are located within the unit interface, and *N*_22_ captures the sorbate–sorbate distribution in the unit interface.

*N*_22_ tends to 0 at low *a*_2_ (Figures 3 and 4) in conformity to Henry’s law. Due to the small contributions from the individual binding of sorbate as can be seen from the small *A*_1_ in Table 1, the sorbate is sparse at low *a*_2_ (Figures 1 and 2), and *N*_22_ is low (Figures 3 and 4), showing a weak sorbate–sorbate correlation in this activity range. At the high end of *a*_2_, *N*_22_ is low (Figures 3 and 4) despite the high population of sorbates as seen from the large ⟨*n*_2_⟩ (Figures 1 and 2). *N*_22_ + 1 tending to 0 reflects the liquid-like nature of sorbates at saturation, which has been inferred from experiments.^89,90^

Previously, we have estimated *N*_22_ + 1 for the PIT-type porous carbons around the peaks of *N*_22_ + 1,^65^ which shows a good agreement with the adsorption lines in Figures 3 and 4, whereas a discrepancy is seen for the desorption lines. Note that the maximum gradient of ⟨*n*_2_⟩ was detected visually without using any fitting model^65^ and that the deviation of our fitting function from the data may have also exaggerated the gradient for the desorption lines.

#### Sorbate–Sorbate Kirkwood–Buff Integral

**Figure 5:**
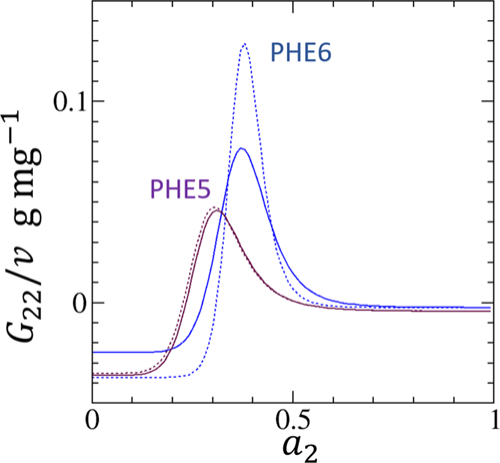
*G*_22_/*v*, where *G*_22_ is the sorbate–sorbate Kirkwood–Buff integral and *v* is the volume of the unit interface, calculated for the adsorption (solid) and desorption (dashed) isotherms of water vapor on PHE5 (maroon) and PHE6 (blue) calculated from eq 21. For the parameters for eq 21, see Figure 1 and Table 1.

**Figure 6:**
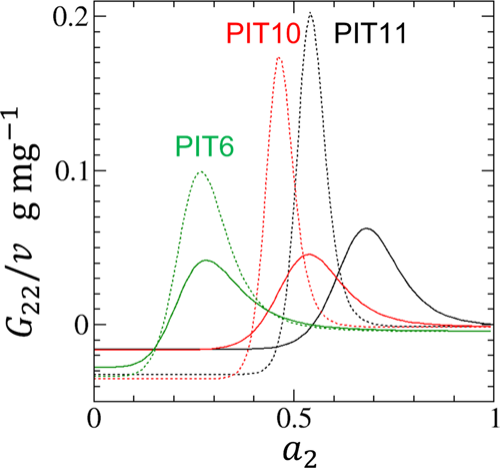
*G*_22_/*v*, where *G*_22_ is the sorbate–sorbate Kirkwood–Buff integral and *v* is the volume of the unit interface, calculated for the adsorption (solid) and desorption (dashed) isotherms of water vapor on PIT6 (green), PIT10 (red), and PIT11 (black), calculated from eq 21. For the parameters for eq 21, see Figure 2 and Table 1.

Sorbate–sorbate interaction, mediated by the interface, determines the *a*_2_ dependence of sorption. How sorbate–sorbate interaction depends on *a*_2_ can be quantified also for the cooperative isotherm using the Kirkwood–Buff integral, *G*_22_, which can be calculated from the following relationship:^70^
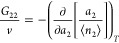
20Combining eqs 20 and 18 yields
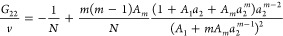
21Note that *v* is proportional to *N*. Here, the extensive variable of ⟨*n*_2_⟩ is usually expressed in an intensive manner, for example, per unit mass of adsorbent. Similarly, ⟨*n*_2_⟩ in eq 20 is reported per unit amount of adsorbent: hence, *G*_22_/*v* in Figures 5 and 6 is reported in the same manner.

We have calculated the *G*_22_/*v* for the experimental isotherm (Figures 5 and 6) of water adsorption on porous carbons, each exhibiting a sharp peak. Such behavior of *G*_22_ and *N*_22_ is the signature of cooperative sorption, different from the adsorption models such as the Langmuir,^34^ BET,^1,35^ and GAB.^36−38^ These models are in fact the special cases of a more general statistical thermodynamic isotherm, founded on the *a*_2_ expansion of the sorbate–sorbate Kirkwood-Buff integral, *G*_22_, as^70^
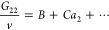
22where the *B* and *C* terms come from the two-body and three-body correlations between sorbates, respectively. We have shown that the generalized isotherm encompassing the three widely used models can be derived from eq 22, via eq 20, as^70^
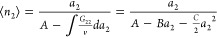
23along with an additional parameter, *A*, from the sorbate–sorbent interaction at the *a*_2_ → 0 limit. A comparison with eq 20 shows the difficulty of capturing cooperative sorption via eq 23 because of the need to incorporate higher-order terms in the *a*_2_ expansion of the Kirkwood–Buff integral (eq 22). See Appendix E for details. Capturing cooperativity directly from eq 18 is conceptually an easier approach, as has been demonstrated.

### Comparison with the Previous Models of Cooperative Sorption

Here we compare our theory with previous isotherm models. Our theory is founded on the generalized Gibbs isotherm applicable to any interfacial geometry and porosity (the ″Theory″ section). The generalized Gibbs isotherm was expressed using the local interfacial subsystem based on the postulate on the finite ranged nature of the interface. The sorption polynomial (eq 9b) was introduced directly from the partition function (the ″Theory″ section). The sorption isotherm (eq 13) derived from the sorption polynomial is a general formula valid as long as the sorbate phase is in vapor. (Generalization of this theory to a solid–liquid interface is conceptually straightforward but mathematically involved due to the presence of terms that can be safely discarded in the treatment in this paper.)

Relationships analogous in form to the sorption polynomial (eq 9b) were derived previously based on various model assumptions. The thermodynamics of ligand binding employs the ″binding polynomial″,^44,91^ which can be expressed as
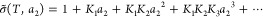
24using the binding constants *K_i_* of successive binding onto a binding site. This binding polynomial is founded on the grand canonical partition function^44,50,52^ and can indeed be derived formally from our sorption isotherm (Appendix B). However, it introduces an assumption that the ligand binding is stoichiometric and successive; these assumptions would need to be confirmed by experiments. The approach based on binding has led to confusion when applied to weak, nonspecific interactions in the solution.^57−59^ Moreover, when applying eq 24 to fit experimental data, various additional assumptions must be incorporated to reduce the number of binding constants or by truncating the expansion.^10,44,91^

A formalism analogous to the binding polynomial is used also in the classical adsorption models. For example, the BET model starts from the following polynomial:
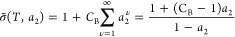
25where *C*_B_ is the BET constant or the binding constant of adsorbate clusters regardless of size.^12,13^ This is equivalent to assuming that the binding of adsorbate *v*-mer on the adsorbent is independent of ν. Further modifications and extensions of eq 25 led to other sorption models, such as the GAB^36−38^ and BDDT^92^ models.

Unlike these models, our theory is derived directly from the principles of statistical thermodynamics. Our approach was motivated by the need to capture the sigmoidal functional shape of the isotherm in which its steep gradient has been attributed to capillary condensation.^13^ As introduced in the ″Theory″ section, the parameters *m*, *A*_1_, and *A_m_* have a clear physical meaning from statistical thermodynamics: *m* is the number of sorbates sorbing cooperatively, and *A*_1_ and *A_m_* are related to the transfer free energy of 1 and *m* sorbates from the saturated vapor (standard state) to the interface, respectively. Our isotherm has a functional form analogous to the (A.V.) Hill isotherm (which is without the *A*_1_ term) yet without the model constructs such as the binding sites and binding constants that may not be convenient for describing capillary condensation. Indeed, no assumptions have been made on the geometry of sorbents or the existence of adsorption sites and layers. The only postulates, in addition to the principles of statistical thermodynamics, were as follows: (i) the effect of the interface is confined within a finite distance; (ii) the interface is extensive; (iii) the sorption isotherm can be described using statistically independent microscopic unit interfaces; (iv) only a few terms (i.e., *a*_2_ and *a*_2_^*m*^ terms) are necessary to capture the cooperative isotherm; and (v) Henry’s law is satisfied at low *a*_2_ limit. We have demonstrated that the isotherm (eq 18) derived from such a set of simple postulates can successfully fit experimental sorption isotherms.

## Conclusions

Adsorption of gases on porous materials often exhibits a sigmoidal isotherm. To understand the molecular interactions underlying such a phenomenon, we have derived an isotherm directly from the principles of statistical thermodynamics. Our foundation was the generalized Gibbs isotherm valid for any interface regardless of surface geometry and porosity. The postulates introduced can be summarized as:

i. the local nature of the interfacial effect (which can be defined through the sorbate-interface distribution function)
ii. the extensive nature of sorption
iii. the existence of a statistically independent unit interface that is microscopic in size
iv. presence of a sharp peak in sorbate number distribution within the unit interface
v. satisfaction of Henry’s law at low activity limit

The first three postulates led to a statistical thermodynamic sorption polynomial, which is a generalization of the binding polynomial and allows a molecular-level interpretation. The latter two led to a simple isotherm function containing the zeroth-, first-, and *m*th-order terms of sorbate activity that are sufficient to capture cooperative sorption. While postulates i, ii, and v are expected to remain valid for all isotherms, we have introduced an approximation in iv, as well as in identifying the unit interface as the statistically independent pore (or pores) in iii. Postulate iv is expected to be insufficient for interfaces with high degrees of structural heterogeneity in which the sorbate number distribution within a unit interface is anticipated to exhibit multiple peaks or broad distributions.

Besides its ability to fit experimental sorption data, our cooperative isotherm, as a generalization of the (A.V.) Hill isotherm and our cooperative solubilization model, has a clear advantage over previous models not only in its direct connection to statistical thermodynamics but also in the clarity in interpretation of their parameters, such as the number of sorbates, *m*, involved in cooperative sorption as well as the transfer free energy of *m* sorbates from the saturated vapor to the interface. This formalism is beneficial in capturing the contribution from capillary condensation. A minor contribution from the sorption of a single sorbate was kept in our theory to satisfy Henry’s law. The sorbate–sorbate Kirkwood–Buff integral, which quantifies sorbate–sorbate interaction, can also be calculated from the isotherm.

In fact, empirical isotherm models may often not give insights on the mechanism of sorption when multiple models based on different assumed adsorption mechanisms can fit the same experimental data. Our theory derives its foundation directly from the principles of statistical thermodynamics. It fills a gap between the statistical thermodynamics of sorption and the analysis of experimental sorption isotherms.

## Appendix A

With the statistically independent units of the interface, now we move on to determining the functional shape of ⟨ν⟩. To do so, let us rewrite the intensive part of the sorption polynomial (eq. 13) into the following form:

A.1

where *P*_ν_is the probability of finding ν sorbates within the unit interface, defined as

A.2

With this setup, let us focus first on the range of *a*_2_ in the cooperative isotherm where ⟨ν⟩ goes through a rapid increase. A large gradient of ⟨ν⟩ indicates (i) a significant probability of the unit interface with no (or very few) sorbates and (ii) *P*_ν_ with a peak at *m* accounting for a sudden increase of ⟨ν⟩, with the width Δν. Neglecting the small contributions outside this width, eq A.1 in this *a*_2_ range becomes

A.3

where we have incorporated *P*_1_ for the low *a*_2_ side to satisfy Henry’s law. In fact, *P*_0_ must also be retained, as

A.4

where, in the numerator, its contribution is 0*P*_0_ = 0.

The presence of *P*_1_ is crucial for satisfying Henry’s law. This can be demonstrated easily by rewriting *P*_ν_ in terms of *A*_ν_ introduced via eq 12, as

A.5

whose first-order term is *A*_1_*a*_2_, which is proportional to the sorbate pressure. This shows that the presence of λ_2_*R̃*_1_ = *a*_2_*A*_1_ in *P*_1_ is the key to satisfying Henry’s law. The neglect of *P*_1_, on the other hand, leads to the disappearance of the first-order term, leading to the violation of Henry’s law. From eq A.5, eq 18 follows straightway.

## Appendix B

Following our previous paper,^65^ we start from the statistical thermodynamic expression for *R*_*n*_2__ in eq 9a, as

B.1

in which *R*(*T*, *V*, *N*_1_, μ_2_; ***X***^*n*_2_^) is defined as^65^

B.2

where Λ*_i_* is the de Broglie wavelength; *q_i_* is the intramolecular partition function; and ***X***^*N*_1_^, ***X***^*N*_2_^′^^, and ***X***^*n*_2_^ denote collectively the coordinates of species 1, species 2 in the bulk (whose number is defined by *N*_2_^′^ = *N*_2_ – *n*_2_), and species 2 in the local subsystem, respectively. Note that the existence of specific binding sites may affect the factor *n*_2_! yet our final isotherm (eq 18) is valid regardless of sorbate identity. Based on this formalism, *R̃**_ν_* for the unit interface (see eq 10) is the scaled-down version of eqs B.1 and B.2 in terms of the volume *v* and particle numbers *n*_2_.

The statistical thermodynamic formalism of *R̃**_ν_* shows that it does not involve any explicit assumptions on successive, stoichiometric binding between *I* (unit *i*nterface) and *S* (sorbate) in the form of

B.3

Equations A.1 and A.2 are not restricted to this assumption. However, it is possible to rewrite *R̃**_ν_*λ_2_^ν^ (or equivalently *A_ν_a*_2_^ν^ via eq 12)

B.4

through which the binding polynomial (eq 24) can be derived from our statistical thermodynamic sorption polynomial (eq 9b). Interpreting the general sorption polynomial (eq 9b) as the binding polynomial (eq 24) using successive stoichiometric constants (eq B.4) is convenient, for example, for ligand binding. However, this picture may be at odds with adsorption on porous materials that involves capillary condensation, as has been discussed in the main text.

## Appendix C

The experimental isotherm is often reported in terms of the ″coverage ratio″, θ, which is defined as

C.1

where *n*_2_^max^ is the maximum adsorption capacity at *a*_2_ → 1. With eq 18, we obtain

C.2

Using eq C.2, the ″coverage ratio″, θ, can be expressed as

C.3

Thus, when the experimental sorption data are reported in terms of θ, eq C.3 should be fitted to the data.

## Appendix D

Usually, the fitting parameters for the cooperative isotherm can be determined by a nonlinear fitting. However, it is useful to consider some limiting cases at which the parameters can be determined from linear regression. The first case is when the sigmoidal increase is the dominant feature in the functional shape of an isotherm. Consequently, the initial rise of the isotherm is negligible, which corresponds to the supposition that the *A*_1_ term is much smaller than the *A_m_* term. Under this condition, eq C.3 can be rewritten as

D.1

Another consequence of the dominance of the *m*th-order term is *A_m_* ≫ 1. Under this condition, eq D.1 leads to

D.2

The parameters *m* and *A_m_* can be determined using a linearized plot of eq D.2, i.e.,

D.3

Accordingly, *m* and *A_m_* can be obtained from the linearized eq D.2 from the isotherm data.

To estimate *A*_1_, we consider the isotherm data at low *a*_2_ where the *a*_2_^*m*^ term is small. Neglecting the terms involving *a*_2_^*m*^,

D.4

Using the condition *A_m_* ≫ *A*_1_ from the previous paragraph, we can approximate eq D.4 as

D.5

where the second approximation involves the Maclaurin expansion. Therefore, *A*_1_ can be estimated using eq D.5 for the isotherm at low *a*_2_.

Thus, we have shown that *A*_1_, *A_m_*, and *m* can be determined using linear regression of experimental data. The parameters determined in this manner can also be used as the input parameters for nonlinear fitting, through which these parameters can be refined.

## Appendix E

Our recent statistical thermodynamic isotherm,^70^ which incorporates the Langmuir,^34^ BET,^1,35^ and GAB^36−38^ models as the special cases, derives the parameters *A*, *B*, and *C* from the *a*_2_ expansions of the Kirkwood–Buff integral. They can be determined via regression using the plot of  against *a*_2_, as follows:

E.1

Here we show that such a plot becomes impractical for cooperative sorption. Rewriting eq 18 in the same format as eq E.1, we obtain

E.2

The Maclaurin expansion of eq E.2 yields

E.3

which shows the presence of *a*_2_, *a*_2_^*m* – 1^, and *a*_2_^*m*^, in contrast to the *a*_2_ and *a*_2_^2^ terms present in eq E.1. We have shown in the ″Results and Discussion″ section that the *a*_2_ term is small in magnitude compared to the cooperativity contribution from the *a*_2_^*m*^ term and that the onset of cooperative adsorption is expressed by the absence of the terms below *a*_2_^*m* – 1^. Therefore, the plot of  against *a*_2_ becomes highly nonlinear. A better fitting strategy, such as the direct nonlinear fitting or the approaches in Appendices C and D, is necessary.

## References

[ref1] Gregg S.; Sing K. S.; Adsorption W., Surface Area, and Porosity; Academic Press: London, 1982; pp. 111–194.

[ref2] Rouquerol F.; Rouquerol J.; Sing K. S. W. Adsorption by Powders and Porous Solids; Elsevier: Amsterdam, 1999; pp. 237–438, 10.1016/b978-0-12-598920-6.x5000-3.

[ref3] Furmaniak S.; Gauden P. A.; Terzyk A. P.; Rychlicki G. Water Adsorption on Carbons - Critical Review of the Most Popular Analytical Approaches. Adv. Colloid Interface Sci. 2008, 137, 82–143. 10.1016/j.cis.2007.08.001. 17919444

[ref4] Liu L.; Tan S. J.; Horikawa T.; Do D. D.; Nicholson D.; Liu J. Water Adsorption on Carbon - A Review. Adv. Colloid Interface Sci. 2017, 250, 64–78. 10.1016/j.cis.2017.10.002. 29129312

[ref5] Do D. D.; Do H. D. Effects of Adsorbate–Adsorbate Interaction in the Description of Adsorption Isotherm of Hydrocarbons in Micro–Mesoporous Carbonaceous Materials. Appl. Surf. Sci. 2002, 196, 13–29. 10.1016/S0169-4332(02)00041-7.

[ref6] Sing K. S. W. Adsorption Methods for the Characterization of Porous Materials. Adv. Colloid Interface Sci. 1998, 76-77, 3–11. 10.1016/S0001-8686(98)00038-4.

[ref7] Kosaka W.; Yamagishi K.; Zhang J.; Miyasaka H. Gate-Opening Gas Adsorption and Host-Guest Interacting Gas Trapping Behavior of Porous Coordination Polymers under Applied Ac Electric Fields. J. Am. Chem. Soc. 2014, 136, 12304–12313. 10.1021/ja504992g. 25120189

[ref8] Foo M. L.; Matsuda R.; Hijikata Y.; Krishna R.; Sato H.; Horike S.; Hori A.; Duan J.; Sato Y.; Kubota Y.; Takata M.; Kitagawa S. An Adsorbate Discriminatory Gate Effect in a Flexible Porous Coordination Polymer for Selective Adsorption of CO2 over C2H2. J. Am. Chem. Soc. 2016, 138, 3022–3030. 10.1021/jacs.5b10491. 26876504

[ref9] Zheng J. J.; Kusaka S.; Matsuda R.; Kitagawa S.; Sakaki S. Theoretical Insight into Gate-Opening Adsorption Mechanism and Sigmoidal Adsorption Isotherm into Porous Coordination Polymer. J. Am. Chem. Soc. 2018, 140, 13958–13969. 10.1021/jacs.8b09358. 30264569

[ref10] Buttersack C. Modeling of Type IV and V Sigmoidal Adsorption Isotherms. Phys. Chem. Chem. Phys. 2019, 21, 5614–5626. 10.1039/c8cp07751g. 30788465

[ref11] Al-Ghouti M. A.; Da’ana D. A. Guidelines for the Use and Interpretation of Adsorption Isotherm Models: A Review. J. Hazard. Mater. 2020, 393, 122383 10.1016/j.jhazmat.2020.122383. 32369889

[ref12] Adamson A. W.; Gast A. P. Physical Chemistry of Surfaces; Wiley: New York, 1997; pp. 599–684.

[ref13] Butt H.-J.; Graf K.; Kappl M. Physics and Chemistry of Interfaces; Wiley-VCH: Weinheim, 2013; pp. 229–265.

[ref14] Sips R. On the Structure of a Catalyst Surface. J. Chem. Phys. 1948, 16, 490–495. 10.1063/1.1746922.

[ref15] Polanyi M. Über die Adsorption vom Standpunkt des Dritten Wärmesatzes. Verh. Dtsch. Phys. Ges 1914, 16, 1012–1016.

[ref16] Polanyi M. Section III.—Theories of the adsorption of gases. A general survey and some additional remarks. Introductory paper to section III. Trans. Faraday Soc. 1932, 28, 316–333. 10.1039/TF9322800316.

[ref17] Polanyi M. The Potential Theory of Adsorption. Science 1963, 141, 1010–1013. 10.1126/science.141.3585.1010. 17739484

[ref18] Frenkel J. Kinetic Theory of Liquids; Clarendon Press: Oxford, 1946; pp. 308–365.

[ref19] Halsey G. Physical Adsorption on Non-Uniform Surfaces. J. Chem. Phys. 1948, 16, 931–937. 10.1063/1.1746689.

[ref20] Hill T. L. Theory of Physical Adsorption. Adv. Catal. 1952, 4, 211–258. 10.1016/S0360-0564(08)60615-X.

[ref21] Sing K. S. W.; Williams R. T. Empirical Procedures for the Analysis of Physisorption Isotherms. Adsorpt. Sci. Technol. 2005, 23, 839–853. 10.1260/026361705777641990.

[ref22] Dubinin M. M.; Astakhov V. A. Development of the Concepts of Volume Filling of Micropores in the Adsorption of Gases and Vapors by Microporous Adsorbents - Communication 2. General Bases of the Theory of Adsorption of Gases and Vapors on Zeolites. Bull. Acad. Sci. USSR Div. Chem. Sci. 1971, 20, 8–12. 10.1007/BF00849308.

[ref23] Dubinin M. M.; Radushkevich L. V. Equation of the Characteristic Curve of Activated Charcoal. Proc. Acad. Sci. USSR, Phys. Chem. Sect. 1947, 55, 331–333.

[ref24] Dubinin M. M. The Potential Theory of Adsorption of Gases and Vapors for Adsorbents with Energetically Nonuniform Surfaces. Chem. Rev. 1960, 60, 235–241. 10.1021/cr60204a006.

[ref25] Dubinin M. M. Physical Adsorption of Gases and Vapors in Micropores. Prog. Surf. Membr. Sci. 1975, 9, 1–70. 10.1016/b978-0-12-571809-7.50006-1.

[ref26] Dubinin M. M. Fundamentals of the Theory of Adsorption in Micropores of Carbon Adsorbents: Characteristics of Their Adsorption Properties and Microporous Structures. Carbon N. Y. 1989, 27, 457–467. 10.1016/0008-6223(89)90078-X.

[ref27] van den Berg C.; Bruin S. Water Activity and Its Estimation in Food Systems: Theoretical Aspects. In Water Activity: Influences on Food Quality; Academic Press: London, 1981; pp. 1–61, 10.1016/b978-0-12-591350-8.50007-3.

[ref28] Avnir D.; Jaroniec M. An Isotherm Equation for Adsorption on Fractal Surfaces of Heterogeneous Porous Materials. Langmuir 1989, 5, 1431–1433. 10.1021/la00090a032.

[ref29] Pfeifer P.; Obert M.; Cole M. W. Fractal BET and FHH Theories of Adsorption: A Comparative Study. Proc. R. Soc. London. A. Math. Phys. Sci. 1989, 423, 169–188. 10.1098/rspa.1989.0049.

[ref30] Pfeifer P.; Wu Y. J.; Cole M. W.; Krim J. Multilayer Adsorption on a Fractally Rough Surface. Phys. Rev. Lett. 1989, 62, 1997–2000. 10.1103/PhysRevLett.62.1997. 10039830

[ref31] Neimark A. V.; Unger K. K. Method of Discrimination of Surface Fractality. J. Colloid Interface Sci. 1993, 158, 412–419. 10.1006/jcis.1993.1273.

[ref32] Bao L.; Ma J.; Long W.; He P.; Zhang T. A.; Nguyen A. V. Fractal Analysis in Particle Dissolution: A Review. Rev. Chem. Eng. 2014, 30, 261–287. 10.1515/revce-2013-0032.

[ref33] Peleg M. Models of Sigmoid Equilibrium Moisture Sorption Isotherms with and without the Monolayer Hypothesis. Food Eng. Rev. 2020, 12, 1–13. 10.1007/s12393-019-09207-x.

[ref34] Langmuir I. The Adsorption of Gases on Plane Surfaces of Glass, Mica and Platinum. J. Am. Chem. Soc. 1918, 40, 1361–1403. 10.1021/ja02242a004.

[ref35] Brunauer S.; Emmett P. H.; Teller E. Adsorption of Gases in Multimolecular Layers. J. Am. Chem. Soc. 1938, 60, 309–319. 10.1021/ja01269a023.

[ref36] Guggenheim E. A. Applications of Statistical Mechanics; Clarendon Press: Oxford, 1966; pp. 186–206.

[ref37] Anderson R. B. Modifications of the Brunauer, Emmett and Teller Equation 1. J. Am. Chem. Soc. 1946, 68, 686–691. 10.1021/ja01208a049. 18861755

[ref38] de Boer J. H. Dynamical Character of Adsorption; Clarendon Press: Oxford, 1968; pp. 200–219.

[ref39] Ward C. A.; Wu J. Effect of Adsorption on the Surface Tensions of Solid-Fluid Interfaces. J. Phys. Chem. B 2007, 111, 3685–3694. 10.1021/jp067066m. 17388534

[ref40] Hill T. L. An Introduction to Statistical Thermodynamics; Dover: New York, 1986; pp. 124–146.

[ref41] Hill A. V. The Possible Effects of the Aggregation of the Molecules of Haemoglobin on Its Dissociation Curves. J. Physiol. 1910, 40, 4–7.

[ref42] Adair G. S.; Bock A. V.; Field H. Jr. The Hemoglobin System. J. Biol. Chem. 1925, 63, 529–545. 10.1016/s0021-9258(18)85018-9.

[ref43] Klotz I. M.; Walker F. M.; Pivan R. B. The Binding of Organic Ions by Proteins 1. J. Am. Chem. Soc. 1946, 68, 1486–1490. 10.1021/ja01212a030. 20994961

[ref44] Klotz I. M. Ligand-Receptor Energetics: A Guide for the Perplexed; Wiley: New York, 1997; pp. 13–32.

[ref45] Koshland D. E.; Nemethy J. G.; Filmer D. Comparison of Experimental Binding Data and Theoretical Models in Proteins Containing Subunits. Biochemistry 1966, 5, 365–385. 10.1021/bi00865a047. 5938952

[ref46] Monod J.; Wyman J.; Changeux J. P. On the Nature of Allosteric Transitions: A Plausible Model. J. Mol. Biol. 1965, 12, 88–118. 10.1016/S0022-2836(65)80285-6. 14343300

[ref47] Wyman J. Linked Functions and Reciprocal Effects in Hemoglobin: A Second Look. Adv. Protein Chem. 1964, 19, 223–286. 10.1016/S0065-3233(08)60190-4. 14268785

[ref48] Wyman J. Heme Proteins. Adv. Protein Chem. 1948, 4, 407–531. 10.1016/S0065-3233(08)60011-X. 18884352

[ref49] Wyman J.; Gill S. J. Binding and Linkage: Functional Chemistry of Biological Macromolecules; University Science Books: Mill Valley, CA, 1990; pp. 33–164.

[ref50] Schellman J. A. Macromolecular Binding. Biopolymers 1975, 14, 999–1018. 10.1002/bip.1975.360140509.

[ref51] Schellman J. A. Solvent Denaturation. Biopolymers 1978, 17, 1305–1322. 10.1002/bip.1978.360170515.

[ref52] Schellman J. A. Selective Binding and Solvent Denaturation. Biopolymers 1987, 26, 549–559. 10.1002/bip.360260408. 3567326

[ref53] Schellman J. A. Fifty Years of Solvent Denaturation. Biophys. Chem. 2002, 96, 91–101. 10.1016/S0301-4622(02)00009-1. 12034431

[ref54] Parsegian V. A.; Rand R. P.; Rau D. C. Macromolecules and Water: Probing with Osmotic Stress. Methods Enzymol. 1995, 259, 43–94. 10.1016/0076-6879(95)59039-0. 8538466

[ref55] Timasheff S. N. In Disperse Solution, “Osmotic Stress” Is a Restricted Case of Preferential Interactions. Proc. Natl. Acad. Sci. U. S. A. 1998, 95, 7363–7367. 10.1073/pnas.95.13.7363. 9636154 PMC22618

[ref56] Parsegian V. A.; Rand R. P.; Rau D. C. Osmotic Stress, Crowding, Preferential Hydration, and Binding: A Comparison of Perspectives. Proc. Natl. Acad. Sci. U. S. A. 2000, 97, 3987–3992. 10.1073/pnas.97.8.3987. 10760270 PMC18129

[ref57] Shimizu S. Estimating Hydration Changes upon Biomolecular Reactions from Osmotic Stress, High Pressure, and Preferential Hydration Experiments. Proc. Natl. Acad. Sci. 2004, 101, 1195–1199. 10.1073/pnas.0305836101. 14732698 PMC337029

[ref58] Shimizu S.; Boon C. L. The Kirkwood-Buff Theory and the Effect of Cosolvents on Biochemical Reactions. J. Chem. Phys. 2004, 121, 9147–9155. 10.1063/1.1806402. 15527383

[ref59] Shimizu S.; Matubayasi N. Preferential Solvation: Dividing Surface vs Excess Numbers. J. Phys. Chem. B 2014, 118, 3922–3930. 10.1021/jp410567c. 24689966

[ref60] Shimizu S. Formulating Rationally via Statistical Thermodynamics. Curr. Opin. Colloid Interface Sci. 2020, 48, 53–64. 10.1016/j.cocis.2020.03.008.

[ref61] Shimizu S.; Matubayasi N. Unifying Hydrotropy under Gibbs Phase Rule. Phys. Chem. Chem. Phys. 2017, 19, 23597–23605. 10.1039/c7cp02132a. 28492648

[ref62] Shimizu S.; Matubayasi N. A Unified Perspective on Preferential Solvation and Adsorption Based on Inhomogeneous Solvation Theory. Phys. A Stat. Mech. its Appl. 2018, 492, 1988–1996. 10.1016/j.physa.2017.11.113.

[ref63] Ribeiro Carrott M. M. L.; Candeias A. J. E.; Carrott P. J. M.; Ravikovitch P. I.; Neimark A. V.; Sequeira A. D. Adsorption of Nitrogen, Neopentane, n-Hexane, Benzene and Methanol for the Evaluation of Pore Sizes in Silica Grades of MCM-41. Microporous Mesoporous Mater. 2001, 47, 323–337. 10.1016/S1387-1811(01)00394-8.

[ref64] Shimizu S.; Matubayasi N. Phase Stability Condition and Liquid–Liquid Phase Separation under Mesoscale Confinement. Phys. A Stat. Mech. its Appl. 2021, 563, 125385 10.1016/j.physa.2020.125385.

[ref65] Shimizu S.; Matubayasi N. Fluctuation Adsorption Theory: Quantifying Adsorbate-Adsorbate Interaction and Interfacial Phase Transition from an Isotherm. Phys. Chem. Chem. Phys. 2020, 22, 28304–28316. 10.1039/D0CP05122E. 33295900

[ref66] Nakamura M.; Ohba T.; Branton P.; Kanoh H.; Kaneko K. Equilibration-Time and Pore-Width Dependent Hysteresis of Water Adsorption Isotherm on Hydrophobic Microporous Carbons. Carbon N. Y. 2010, 48, 305–308. 10.1016/j.carbon.2009.09.008.

[ref67] Gibbs J. W. The Collected Works of J. W. Gibbs; Yale University Press: New Haven, CT, 1928; pp. 219–237.

[ref68] Scatchard G. The Gibbs Adsorption Isotherm 1. J. Phys. Chem. 1962, 66, 618–620. 10.1021/j100810a011.

[ref69] Defay R.; Prigogine I. Tension Superficielle et Adsorption; Desoer: Liege, 1966; pp. 71–79.

[ref70] Shimizu S.; Matubayasi N. Sorption: A Statistical Thermodynamic Fluctuation Theory. Langmuir 2021, 37, 7380–7391. 10.1021/acs.langmuir.1c00742. 34124912 PMC8280703

[ref71] Giles C. H.; Smith D.; Huitson A. A General Treatment and Classification of the Solute Adsorption Isotherm. I. Theoretical. J. Colloid Interface Sci. 2016, 47, 755–765. 10.1007/s41193-016-0111-5.

[ref72] Madani S. H.; Kwong P.; Rodríguez-Reinoso F.; Biggs M. J.; Pendleton P. Decoding Gas-Solid Interaction Effects on Adsorption Isotherm Shape: I. Non-Polar Adsorptives. Microporous Mesoporous Mater. 2018, 264, 76–83. 10.1016/j.micromeso.2018.01.010.

[ref73] Madani S. H.; Biggs M. J.; Rodríguez-Reinoso F.; Pendleton P. Decoding Gas-Solid Interaction Effects on Adsorption Isotherm Shape: II. *Polar Adsorptives*. Microporous Mesoporous Mater. 2019, 278, 232–240. 10.1016/j.micromeso.2018.11.039.

[ref74] Horikawa T.; Do D. D.; Nicholson D. Capillary Condensation of Adsorbates in Porous Materials. Adv. Colloid Interface Sci. 2011, 169, 40–58. 10.1016/j.cis.2011.08.003. 21937014

[ref75] Shimizu S.; Matubayasi N. Hydrotropy: Monomer-Micelle Equilibrium and Minimum Hydrotrope Concentration. J. Phys. Chem. B 2014, 118, 10515–10524. 10.1021/jp505869m. 25144510

[ref76] Nicol T. W. J.; Matubayasi N.; Shimizu S. Origin of Non-Linearity in Phase Solubility: Solubilisation by Cyclodextrin beyond Stoichiometric Complexation. Phys. Chem. Chem. Phys. 2016, 18, 15205–15217. 10.1039/C6CP01582D. 27206059

[ref77] Shimizu S.; Matubayasi N. Hydrotropy and Scattering: Pre-Ouzo as an Extended near-Spinodal Region. Phys. Chem. Chem. Phys. 2017, 19, 26734–26742. 10.1039/c7cp04990k. 28948253

[ref78] Do D. D.; Do H. D. A Model for Water Adsorption in Activated Carbon. Carbon N. Y. 2000, 38, 767–773. 10.1016/S0008-6223(99)00159-1.

[ref79] Do D. D.; Junpirom S.; Do H. D. A New Adsorption-Desorption Model for Water Adsorption in Activated Carbon. Carbon N. Y. 2009, 47, 1466–1473. 10.1016/j.carbon.2009.01.039.

[ref80] Rutherford S. W. Modeling Water Adsorption in Carbon Micropores: Study of Water in Carbon Molecular Sieves. Langmuir 2006, 22, 702–708. 10.1021/la051826n. 16401120

[ref81] Shimizu S.; Matubayasi N. The Origin of Cooperative Solubilisation by Hydrotropes. Phys. Chem. Chem. Phys. 2016, 18, 25621–25628. 10.1039/C6CP04823D. 27711657

[ref82] Soares B. P.; Abranches D. O.; Sintra T. E.; Leal-Duaso A.; García J. I.; Pires E.; Shimizu S.; Pinho S. P.; Coutinho J. A. P. Glycerol Ethers as Hydrotropes and Their Use to Enhance the Solubility of Phenolic Acids in Water. ACS Sustainable Chem. Eng. 2020, 8, 5742–5749. 10.1021/acssuschemeng.0c01032.

[ref83] Abranches D. O.; Benfica J.; Soares B. P.; Leal-Duaso A.; Sintra T. E.; Pires E.; Pinho S. P.; Shimizu S.; Coutinho J. A. P. Unveiling the Mechanism of Hydrotropy: Evidence for Water-Mediated Aggregation of Hydrotropes around the Solute. Chem. Commun. 2020, 56, 7143–7146. 10.1039/d0cc03217d. 32462150

[ref84] Matubayasi N.; Shinoda W.; Nakahara M. Free-Energy Analysis of the Molecular Binding into Lipid Membrane with the Method of Energy Representation. J. Chem. Phys. 2008, 128, 195107 10.1063/1.2919117. 18500905

[ref85] McMillan W. G. Jr.; Mayer J. E. The Statistical Thermodynamics of Multicomponent Systems. J. Chem. Phys. 1945, 13, 276–305. 10.1063/1.1724036.

[ref86] Landau L. D.; Lifshitz E. M. Statistical Physics, 3rd Edition, Part I; Pergamon Press: London, 1986; pp. 111–124.

[ref87] Shimizu S.; Matubayasi N. Intensive Nature of Fluctuations: Reconceptualizing Kirkwood-Buff Theory via Elementary Algebra. J. Mol. Liq. 2020, 318, 114225 10.1016/j.molliq.2020.114225.

[ref88] Kadlec O. The History and Present State of Dubinin’s Theory of Adsorption of Vapours and Gases on Microporous Solids. Adsorpt. Sci. Technol. 2001, 19, 1–24. 10.1260/0263617011493944.

[ref89] Nguyen C.; Do D. D. The Dubinin-Radushkevich Equation and the Underlying Microscopic Adsorption Description. Carbon N. Y. 2001, 39, 1327–1336. 10.1016/S0008-6223(00)00265-7.

[ref90] Shimizu S.; Matubayasi N. Adsorbate-Adsorbate Interactions on Microporous Materials. Microporous Mesoporous Mater. 2021, 323, 111254 10.1016/j.micromeso.2021.111254.

[ref91] Klotz I. M. Protein Interactions with Small Molecules. Acc. Chem. Res. 1974, 7, 162–168. 10.1021/ar50077a006.

[ref92] Brunauer S.; Deming L. S.; Deming W. E.; Teller E. On a Theory of the van der Waals Adsorption of Gases. J. Am. Chem. Soc. 1940, 62, 1723–1732. 10.1021/ja01864a025.

